# Liver fibrosis staging by deep learning: a visual-based explanation of diagnostic decisions of the model

**DOI:** 10.1007/s00330-021-08046-x

**Published:** 2021-05-20

**Authors:** Yunchao Yin, Derya Yakar, Rudi A. J. O. Dierckx, Kim B. Mouridsen, Thomas C. Kwee, Robbert J. de Haas

**Affiliations:** 1 Department of Radiology, Medical Imaging Center Groningen, University of Groningen, University Medical Center Groningen, PO Box 30001, 9700 RB Groningen, The Netherlands; 2 Department of Clinical Medicine – Center of Functionally Integrative Neuroscience, Aarhus University, Aarhus, Denmark

**Keywords:** Deep learning, Liver cirrhosis, Tomography, X-ray computed, Neural networks, computer, Algorithms

## Abstract

**Objectives:**

Deep learning has been proven to be able to stage liver fibrosis based on contrast-enhanced CT images. However, until now, the algorithm is used as a black box and lacks transparency. This study aimed to provide a visual-based explanation of the diagnostic decisions made by deep learning.

**Methods:**

The liver fibrosis staging network (LFS network) was developed at contrast-enhanced CT images in the portal venous phase in 252 patients with histologically proven liver fibrosis stage. To give a visual explanation of the diagnostic decisions made by the LFS network, Gradient-weighted Class Activation Mapping (Grad-cam) was used to produce location maps indicating where the LFS network focuses on when predicting liver fibrosis stage.

**Results:**

The LFS network had areas under the receiver operating characteristic curve of 0.92, 0.89, and 0.88 for staging significant fibrosis (F2–F4), advanced fibrosis (F3–F4), and cirrhosis (F4), respectively, on the test set. The location maps indicated that the LFS network had more focus on the liver surface in patients without liver fibrosis (F0), while it focused more on the parenchyma of the liver and spleen in case of cirrhosis (F4).

**Conclusions:**

Deep learning methods are able to exploit CT-based information from the liver surface, liver parenchyma, and extrahepatic information to predict liver fibrosis stage. Therefore, we suggest using the entire upper abdomen on CT images when developing deep learning–based liver fibrosis staging algorithms.

**Key Points:**

• *Deep learning algorithms can stage liver fibrosis using contrast-enhanced CT images, but the algorithm is still used as a black box and lacks transparency*.

• *Location maps produced by Gradient-weighted Class Activation Mapping can indicate the focus of the liver fibrosis staging network*.

• *Deep learning methods use CT-based information from the liver surface, liver parenchyma, and extrahepatic information to predict liver fibrosis stage*.

**Supplementary Information:**

The online version contains supplementary material available at 10.1007/s00330-021-08046-x.

## Introduction

In patients with liver fibrosis, normal liver parenchyma is replaced by scar tissue [1]. Main causes of liver fibrosis are excessive alcohol use, severe steatosis/steatohepatitis, and viral hepatitis [2]. Cirrhosis is the most severe stage of liver fibrosis, which can lead to portal hypertension, development of hepatocellular carcinoma, and liver failure. Therefore, it is important to adequately diagnose and stage liver fibrosis before its progression into irreversible, end-stage liver disease. The current gold standard for liver fibrosis diagnosis and staging is histopathological examination of liver tissue obtained through percutaneous biopsy. However, biopsy has several drawbacks, such as peri-procedural pain or discomfort, major hemorrhage with a reported mortality rate up to 1.6%, and the risk of sampling error due to analysis of only a small liver parenchyma specimen [3–5].

To overcome these disadvantages, non-invasive imaging-based methods have been explored to replace biopsy [6, 7]. Several studies have shown deep learning methods to be able to stage liver fibrosis on CT or MRI images [8–10]. However, lack of transparency and explainability (black box principle) can be considered barriers towards clinical acceptance and implementation in standard practice. It is currently unclear whether the convolutional neural network (CNN) is making diagnostic decisions based on established radiological morphologic features related to liver fibrosis (e.g., enhancing fibrotic septa, parenchymal changes including regenerative or dysplastic nodules, and signs of portal hypertension) [6, 7], or whether it uses other features or information [11]. To be able to trust the diagnostic decisions made by deep learning methods, it is important to understand how the system works. Although understanding deep learning methods is still a technical challenge, algorithms have been developed to visualize the region the network focuses on when making predictions [12–14]. Gradient-weighted Class Activation Mapping (Grad-cam) is a state-of-the-art technique producing localization maps highlighting important regions, thereby providing visual explanations for model decisions [12]. Because liver fibrosis can be considered a systemic disease, we hypothesize that for deep learning techniques, information outside the liver might be just as important as the liver itself. This might further increase clinicians’ trust in deep learning methods.

To test our hypothesis, this study aimed to provide a visual-based explanation of the diagnostic decisions made by deep learning in predicting liver fibrosis stages on abdominal CT scans by using Grad-cam.

## Materials and methods

This study is a retrospective and exploratory study investigating the explainability of the LFS network. This study was approved by the local institutional review board (number: 2018/139), and the need for obtaining informed consent was waived.

### Study population

**Fig. 1:**
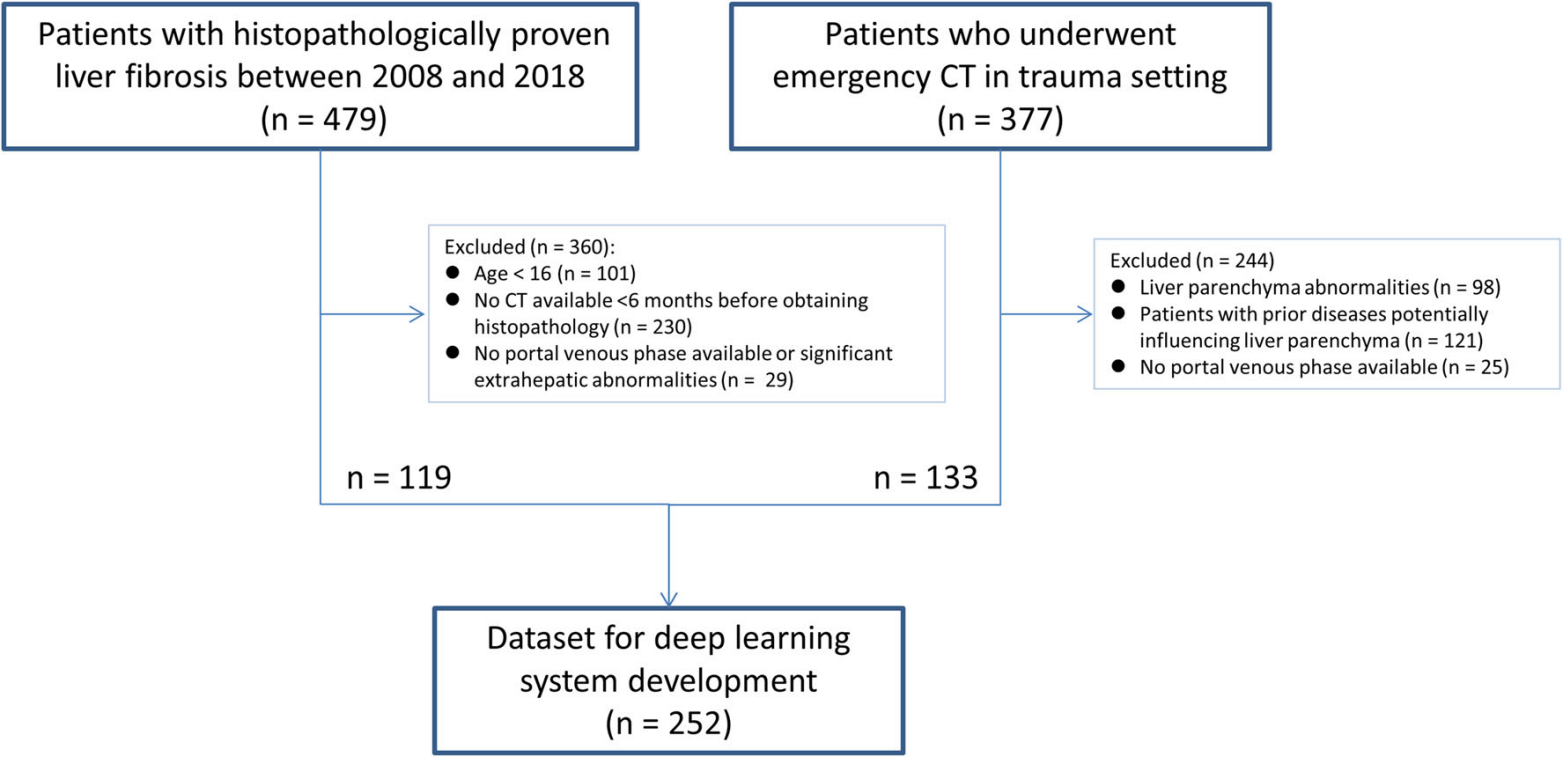
Flowchart of patient inclusion. Abbreviation: CT = computed tomography

Patients treated at our tertiary referral center between 2006 and 2018 who fulfilled the following criteria were included in the study (Fig. 1): (i) age ≥ 16 years; (ii) availability of a contrast-enhanced CT scan in the portal venous phase without relevant extrahepatic abnormalities and displaying the complete liver; (iii) availability of histopathological proof of the degree of liver fibrosis, either through liver biopsy, liver resection, or after liver transplantation; (iv) time interval between CT imaging and obtaining histopathology < 6 months.

To obtain a sufficient number of patients without liver fibrosis (in whom generally no liver tissue samplings are performed), a patient group was added in whom during the same inclusion period a contrast-enhanced abdominal CT scan in the portal venous phase was performed at the emergency department to rule out traumatic injuries. These patients were included in case of absence of liver injuries or other morphologic abnormalities of the liver, and when there was no history of liver disease or associated risk factors. Subsequently, the inclusion criteria were checked on each CT scan by board-certified abdominal radiologists.

Of each patient, the following clinical variables were collected: birth date, gender, cause of liver fibrosis, type of histopathological specimen, relevant medical history, (prior) use of systemic chemotherapy, and tumor markers including alpha-fetoprotein, carbohydrate antigen 19-9, and carcinoembryonic antigen levels.

### Reference standard generation

The liver fibrosis stage was determined by a specialized liver pathologist by using a staging system consisting of 5 stages (stage 0, healthy liver without fibrosis; stage 1, fibrosis of portal area without septa; stage 2, portal fibrosis with few septa; stage 3, septal fibrosis without cirrhosis; stage 4, cirrhosis) [15].

### Algorithm scheme

**Fig. 2:**
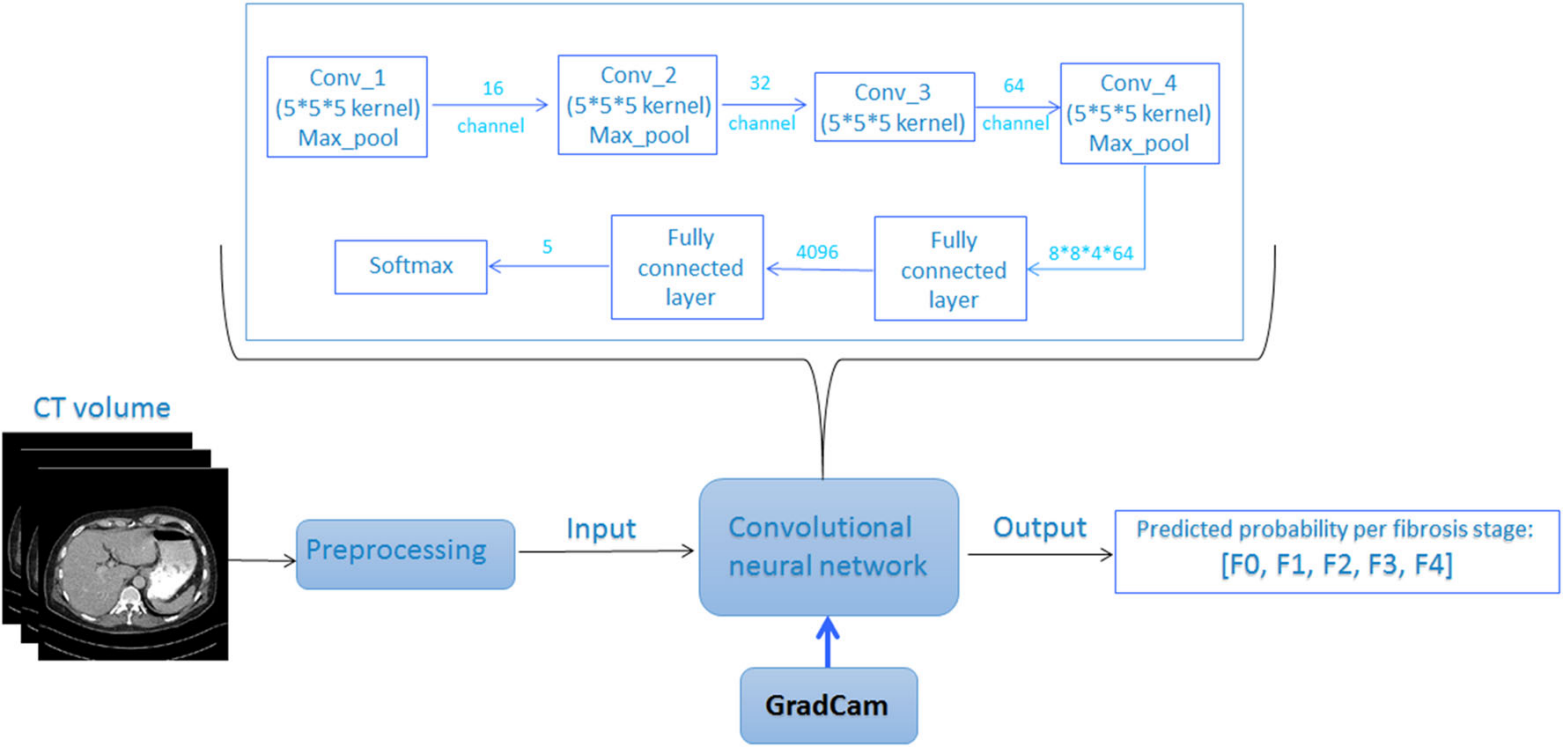
Overall scheme of liver fibrosis staging by deep learning. The computed tomography (CT) scan was first pre-processed by tissue windowing and standardized to [0,1]. Then, 32 consecutive slices of the 3D segmented liver were randomly selected as a patch per training iteration to feed into the convolutional neural network. The network put out the array of predicted probabilities at fibrosis stage (F0–F4). During testing of the trained liver fibrosis staging network, Grad-Cam was integrated between the third convolutional layer and the final convolutional layer to generate the maps showing the location of the network’s focus. Abbreviations: CT: computed tomography; Conv: convolutional layer; Max_pool: maximum pooling layer; GradCam: Gradient-weighted Class Activation Mapping; 5*5 kernel: a kernel with size [5] is used to extract features in the convolutional layer; channel: number of kernels applied in between convolutional layers; Softmax: softmax activation function

To investigate the importance of extrahepatic structures when predicting liver fibrosis stages, we built the LFS network and trained it with abdominal CT volumes. Grad-cam was integrated between the final convolutional layers to visualize which abdominal region the network is focusing on. Subsequently, the reliability of the LFS network can be verified by the radiologist by checking whether the highlighted abdominal regions are clinically relevant for liver fibrosis staging. The overall scheme is shown in Fig. 2.

First, the CT scans were pre-processed. The density values on the original CT scan ranged from −1024 to 3071 Hounsfield units (HU), but the density of abdominal tissue only concerns a small part of the full range. Therefore, we applied a soft tissue window ranging between −125 and +225 HU on the input CT slices to remove irrelevant information and enhance contrast among abdominal organs.

The processed CT scans were then passed to the LFS network consisting of four convolutional layers, three pooling layers, and two fully connected layers. The final fully connected layer puts out the predicted probabilities of each liver fibrosis stage. Each convolutional layer has multiple channels to extract different features from the original input, and the feature maps keep the spatial information from the input CT volume. The common practice is to interpret the network based on this remained spatial information [12–14]. Grad-cam assigns weights to each feature map, which indicate the degree of contribution of the features to the diagnostic decision. The weight of each voxel on the location map represents the importance that the LFS network assigned to the location on the input CT. Higher weights on the location maps indicate a higher degree of attention of the LFS network to the specific area on the corresponding input CT. By visualizing the map generated by Grad-cam, spatial information of features contributing to the diagnostic decision can be obtained. More details regarding the training of the LFS network can be found in Appendix 1.

The entire project was programmed in Python 3.0. Simple-Insight Toolkit and Numpy libraries were used during pre-processing [16, 17], and the LFS network was constructed under the Tensorflow framework, which is an open-source library for deep learning [18].

### Evaluation metrics

The evaluation metrics consisted of two parts: the evaluation metrics of liver fibrosis staging, and the metrics to analyze the location maps indicating the regions the network focuses on.

Five-fold cross-validation was applied to evaluate the generalization ability of the trained LFS network. Similar to other liver fibrosis staging studies [9, 10], the receiver operating characteristic (ROC) curve of significant fibrosis (stages F2, F3, and F4), advanced fibrosis (stages F3 and F4), and cirrhosis (stage F4) was plotted. The sensitivity, specificity, accuracy, and area under the ROC curve (AUC) were used to evaluate the LFS network performance. Besides, micro- and macro-average ROC curves were plotted to evaluate the LFS network performance as a multi-stage classification. The macro-average ROC curve reduces the F0–F4 stages’ classification to multiple sets of binary classifications, while a micro-average ROC curve averages each sample for an aggregate result.

The focus patterns on the location maps generated by Grad-cam were assessed through visual inspection by three radiologists. To quantify the value of the liver parenchyma on the location maps, a box plot of the mean value in the segmented liver area on the Grad-cam location maps was generated.

The statistical analyses were performed in Python 3.0. The ROC curves were plotted and the AUCs were calculated based on scikit-learn library [19].

## Results

### Study population

A total number of 252 patients were included in the study to develop the LFS network. The number of patients categorized according to liver fibrosis stage is shown in Table 1. Two patients who underwent a liver transplantation were included twice in the dataset, as the histopathology results were based on different livers (i.e., the native liver before transplantation and the transplanted liver after re-transplantation).

**Table 1:** Demographics of the study population

Variable		Total cohort	Liver fibrosis stage				
			F0	F1	F2	F3	F4
Total number of patients		252	134	8	10	18	82
Median age (interquartile range)		59 (48–65)	63 (50–74)	64 (38–71)	57 (43–64)	48 (43–62)	60 (54–65)
Gender	Male	140 (55.6%)	68 (50.7%)	3 (37.5%s)	7 (70.0%)	11 (61.1%)	51 (62.2%)
	Female	112 (44.4%)	66 (49.3%)	5 (62.5%)	3 (30.0%)	7 (38.9%)	31 (37.8%)
Origin of histopathology specimen	Biopsy	39 (33.1%)	-	7	6	6	17
	Resection	5 (4.2%)	-	0	0	0	5
	Transplantation	74 (62.7%)	-	1	4	12	57
Etiology of liver fibrosis	Alcoholic	26 (22.0%)	-	0	0	1	25
	Autoimmune hepatitis	5 (4.2%)	-	0	1	1	3
	HBV	3 (2.5%)	-	0	0	0	3
	HCV	10 (8.5%)	-	0	0	1	9
	PSC	3 (2.5%)	-	0	2	1	0
	Steatohepatitis	8 (6.8%)	-	0	0	0	8
	Wilson disease	1 (0.8%)	-	0	0	0	1
	Other	17 (14.4%)	-	1	0	7	9
	Unknown	45 (38.1)	-	7	7	7	24

Abbreviations: HBV = hepatitis B virus; HCV = hepatitis C virus; PSC = primary sclerosing cholangitis

### LFS network accuracy on the test set

**Fig. 3:**
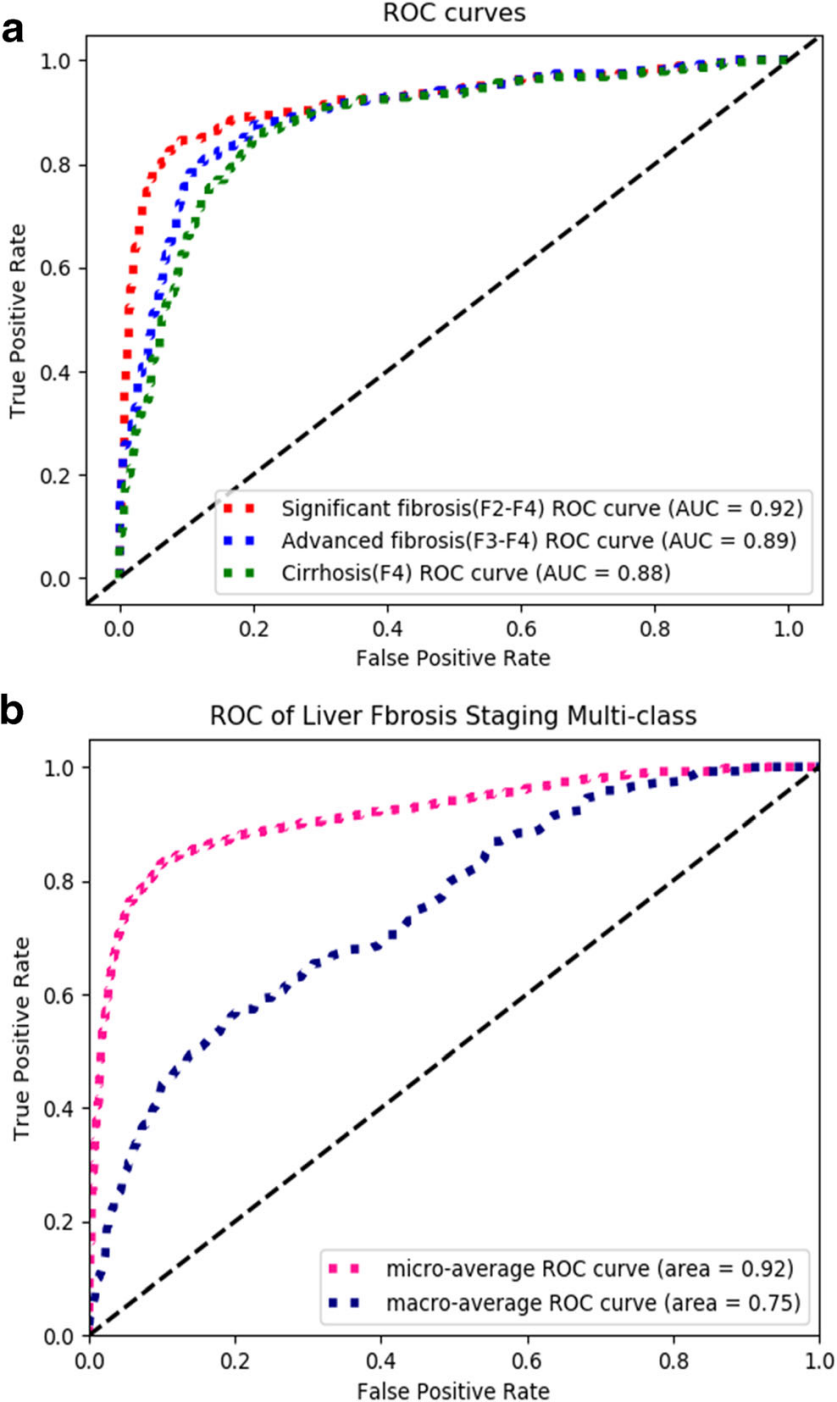
Receiver operating characteristic (ROC) curves of the test sets. **a** ROC curves of the predicted liver fibrosis severity on the test sets, including significant fibrosis, advanced fibrosis, and cirrhosis. **b** Macro-averaged ROC curve reducing the F0–F4 stages’ classification to multiple sets of two-class classifications, and micro-averaged ROC curve averaging each sample of an aggregate result

The AUCs of staging significant fibrosis, advanced fibrosis, and cirrhosis were 0.92, 0.89, and 0.88, respectively, on the test set, and the corresponding ROC curves can be found in Fig. 3. The specificity, sensitivity, and accuracy values are listed in Table 2.

**Table 2:** Summary of the performance of the liver fibrosis staging network in predicting liver fibrosis severity in the test set. The corresponding receiver operating characteristic curves can be found in Fig. 3

	Significant fibrosis (F2–F4)	Advanced fibrosis (F3–F4)	Cirrhosis (F4)
AUC	0.92 [0.86, 0.97]	0.89 [0.83, 0.96]	0.88 [0.79, 0.94]
Specificity (%; 95% CI)	91.7 [82.0, 96.8]	88.2 [77.8, 95.6]	86.5 [78.9, 94.4]
Sensitivity (%; 95% CI)	83.0 [71.7, 94.7]	79.5 [68.2, 92.5]	75.1 [56.5, 86.7]
Accuracy (%; 95% CI)	88.3 [81.5, 94.5]	85.2 [77.0, 92.0]	83.3 [76.2, 90.0]

Abbreviations: AUC = area under the curve; CI = confidence interval

### Location map analysis

**Fig. 4:**
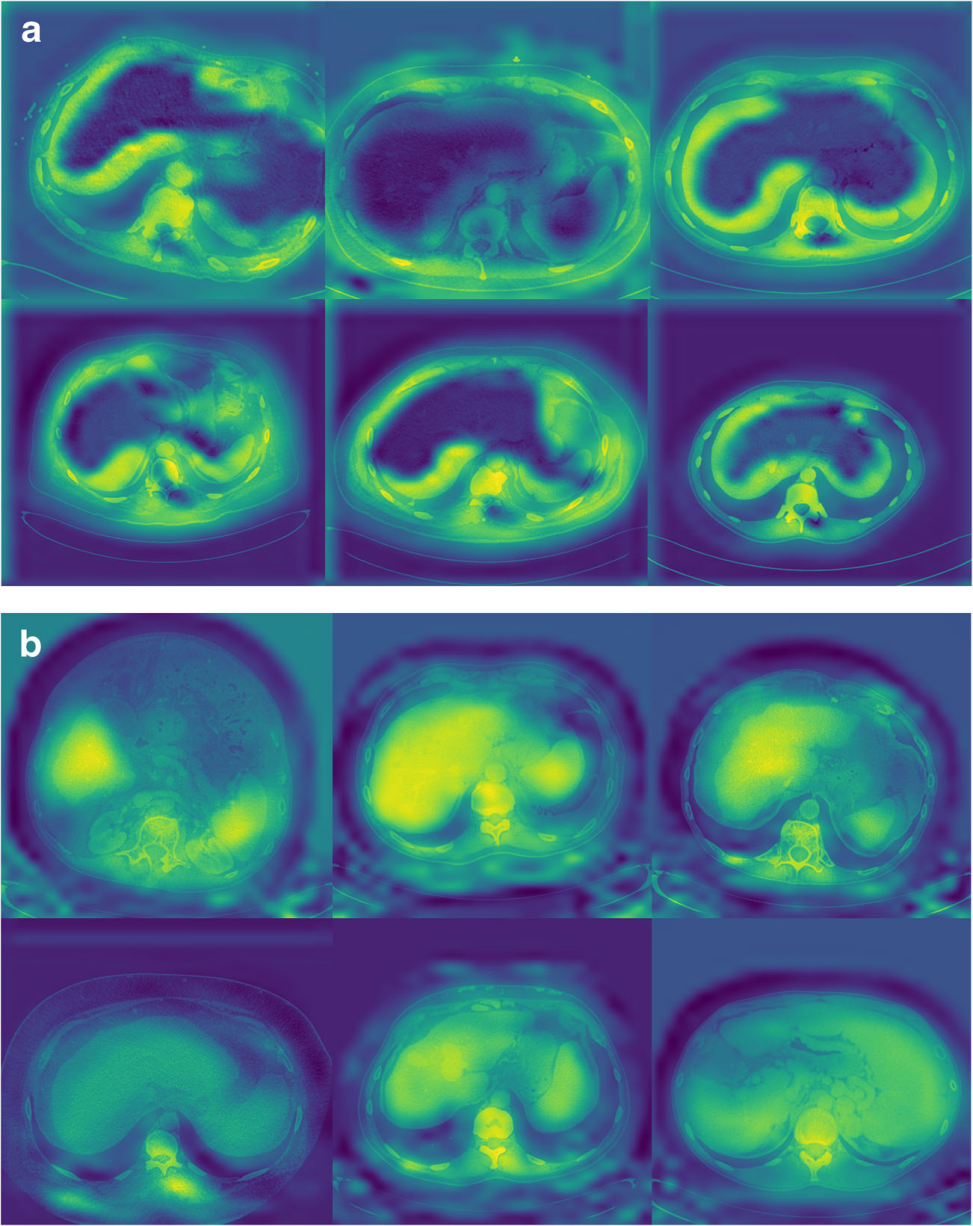
**a** Shown are location maps overlaid on axial computed tomography images of the upper abdomen at different levels in patients without liver fibrosis (stage F0). The liver surface is highlighted in these location maps, which indicates that information exploited from the liver surface contributed to the convolutional neural network's prediction of F0 liver fibrosis. **b** Shown are location maps overlaid on axial computed tomography images of the upper abdomen at different levels in patients with cirrhosis (stage F4). The liver parenchyma and spleen are highlighted in these location maps, which indicates that information exploited from the liver parenchyma and spleen contributed to the convolutional neural network’s prediction of F4 liver fibrosis (cirrhosis)

As mentioned before, the location maps indicate the areas contributing to the diagnostic decisions made by the LFS network. Two patterns on the location maps were identified: (i) when the LFS network prediction is based on a CT scan from patients with a healthy liver (fibrosis stage F0), the surface of the liver is highlighted on the location maps, but the central area of the liver parenchyma is not; this pattern was observed in 91.0% of the location maps in patients with F0 liver fibrosis; (ii) when the LFS network prediction is based on a CT scan from patients with cirrhosis (fibrosis stage F4), both the liver and spleen (mainly central areas) are highlighted; this pattern was observed in 76.4% of the location maps in patients with cirrhosis (F4 liver fibrosis). Several examples are shown in Fig. 4.

**Fig. 5:**
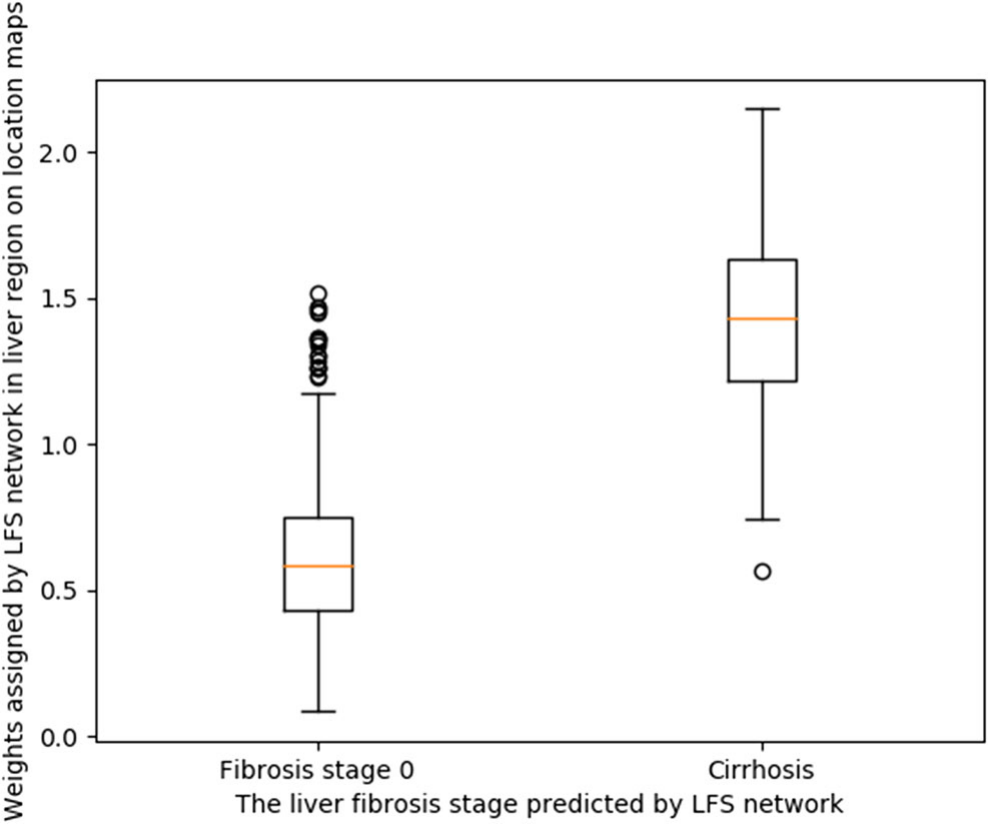
Distribution of mean weights assigned by the liver fibrosis staging (LFS) network in the liver parenchyma on the location maps. The weights represent the importance of the liver parenchyma on CT for the LFS network when making diagnostic decisions. The two groups are divided according to the liver fibrosis stage predicted by the LFS network

In a post hoc analysis to further quantify the observed patterns in the location maps, we selected the liver parenchyma as region of interest, and calculated the average weights assigned by the LFS network in the region of interest on the location maps, with higher values indicating greater attention of the network when predicting liver fibrosis stages. The liver parenchyma was segmented by the fine-tuned V-net with 0.99-pixel accuracy and 0.90 F1 score (dice coefficient). The median and interquartile range of weights in the liver parenchyma on the location maps were 0.58 (0.43–0.75) and 1.43 (1.22–1.63) for F0 and F4 liver fibrosis, respectively. The difference between the distribution of weights in the liver parenchyma on the location maps was statistically significant (*p* < 0.001). The distribution of two groups of weights is illustrated in the box plot in Fig. 5.

## Discussion

The aim of this study was to improve the explainability of the liver fibrosis staging neural network. This was done by developing deep learning methods to stage liver fibrosis followed by applying Grad-Cam to determine the region of interest of the network when predicting the liver fibrosis stage of the test set. By doing so, two patterns of regions of interest of the neural network in the upper abdomen could be differentiated, namely a highlighted liver surface on the location maps in case of F0 liver fibrosis, versus highlighted central areas in the liver and spleen in patients with F4 liver fibrosis.

The diagnostic accuracy rates of predicting significant fibrosis, advanced fibrosis, and cirrhosis were 88.3%, 85.2%, and 83.2%, respectively, and the AUCs were 0.92, 0.89, and 0.88. These diagnostic performance results are comparable to several previous studies which investigated the possibilities of deep learning to determine liver fibrosis stage. Yasaka et al [10] trained a liver fibrosis staging CNN by cropped liver patches on CT scans from 286 patients, leading to AUCs for determining significant fibrosis, advanced fibrosis, and cirrhosis of 0.74, 0.76, and 0.73, respectively. Instead of cropped patches, Choi et al [8] developed a liver fibrosis staging CNN by using the segmented liver and evaluated the CNN with a multicenter test dataset consisting of 6670 patients. The AUCs for diagnosing significant fibrosis, advanced fibrosis, and cirrhosis reached 0.96, 0.97, and 0.95, respectively [8].

Although deep learning methods have been shown to be able to stage liver fibrosis based on CT images in both studies, it is still unknown which part of the CT image is used by the neural network for liver fibrosis staging. Also, it is unclear whether the available information outside the liver in the upper abdomen contributes to the diagnostic decisions made by the neural network. Location maps illustrating the spatial information of the areas contributing to the fibrosis stage prediction can provide further explanation of the diagnostic decisions made by the neural network. In the current study, we observed that the neural network focused not only on liver parenchyma but also on other information available on CT scans of the upper abdomen when predicting liver fibrosis stages, such as information derived from the liver and spleen. When the network decided that it concerned a healthy liver, the corresponding location map showed that the network strongly focused on the liver surface instead of liver parenchyma, while in the case of cirrhosis (F4 liver fibrosis), the network paid more attention to the central parts of the liver parenchyma and spleen. The box plot of weights assigned by the LFS network in liver parenchyma for F0 and F4 further supports our observations. Therefore, we believe the neural network should be trained on CT slices including the complete upper abdomen, instead of CT images including only the segmented liver. From a clinical point of view, it seems logical that in the case of cirrhosis the neural network focuses both on the liver and spleen, because both organs undergo morphologic changes when cirrhosis develops (i.e., fibrotic changes in the liver parenchyma, increased nodularity of the liver, splenomegaly, and splenic siderotic nodules) [20–22]. To our knowledge, this is the first study giving more insight into decisions taken by deep learning techniques when staging liver fibrosis.

Knowing that the neural network uses information of the liver surface, liver parenchyma, and spleen on CT images when staging liver fibrosis, improves the transparency and interpretability of the network’s prediction of liver fibrosis stages. The location maps can also be used as quality control of the network’s fibrosis stage prediction. For example, physicians can reconsider the network’s prediction of liver fibrosis stages when the location maps show an abnormal pattern or when these maps focus on regions not considered to be related to liver fibrosis. Besides, it further underlines that extrahepatic signs of liver fibrosis should be taken into consideration both in clinical practice and when developing liver fibrosis staging software.

Our study had some limitations. First, the training dataset in our study was relatively small. Because deep learning methods are data-driven, diagnostic accuracy and stability of location maps highly depend on the variety and volume of the training set. Therefore, our results should be confirmed in larger (multicenter) studies. Second, the datasets of different fibrosis stages were imbalanced, due to the fact that patients with stage 1 or 2 liver fibrosis generally do not undergo liver biopsy, and thus lack a reliable reference standard. This was also observed in the studies of Yasaka et al [9, 10] and Choi et al [8]. Finally, often, only the upper part of the abdomen was available at the CT images, related to the indication for the CT scan. Perhaps, CT images of the entire abdomen could provide more information, thereby potentially improving deep learning performance. This should be the topic of further research.

In conclusion, the neural network for liver fibrosis staging exploits CT-based information from the liver surface, liver parenchyma, and spleen. Using the entire upper abdomen instead of the segmented liver improves the development of liver fibrosis staging algorithms. In addition, the application of Grad-cam increases transparency, and thereby reliability, of the liver fibrosis staging neural network by offering a visual explanation of information used by the network. Our results might also serve as a roadmap to unravel the black box principle of artificial intelligence in image analysis tasks in other medical fields.

## Supplementary information

ESM 1 (DOCX 17 kb)

## Abbreviations

AUC: Area under the receiver operating characteristic curve
CNN: Convolutional neural network
Grad-cam: Gradient-weighted Class Activation Mapping
HU: Hounsfield units
LFS network: Liver fibrosis staging network
ROC: Receiver operating characteristic

## Declarations

### Guarantor

The scientific guarantor of this publication is Dr. Robbert J. de Haas.

### Statistics and Biometry

No complex statistical methods were necessary for this paper.

### Methodology

• Retrospective

• observational

• performed at one institution
