# Supplementary material for: Liver fibrosis staging by deep learning: a visual-based explanation of diagnostic decisions of the model
Source: Eur Radiol. 2021 May 20;31(12):9620–7. doi: 10.1007/s00330-021-08046-x (PMC8589780; doi:10.1007/s00330-021-08046-x)
Supplement: Supplementary file 1 — (DOCX 17 kb) [file 330_2021_8046_MOESM1_ESM.docx]

# APPENDIX 1

# *Artificial intelligence model training*

The LFS network was trained by the dataset originating from our hospital as described earlier. To mitigate the need for extensive computational power requirements, we randomly took a patch consisting of 32 CT slices per training iteration to feed into the LFS network. To allow the network to make full use of the training data, the CT volume needs to be fed into the network repeatedly (one cycle through the entire training set is called an epoch). The best performing model was selected at the 40th, 54th, 38th, 42th, and 40th epoch at each fold by the validation set consisting of 8 CT scans.

To evaluate the location maps in the following sections, we trained a V-net for liver segmentation [1]. The V-net was first trained by two public datasets, IRCADB (https://www.ircad.fr/research/3d-ircadb-01/) and Sliver07 (https://sliver07.grand-challenge.org/). Each public dataset contained 20 CT scans from different patients, as well as the corresponding label of the segmented liver. Further details of the public dataset can be found on their official websites. The pre-model with best performance was selected at the 58th epoch by the validation set consisting of 5 CT scans from our hospital. However, the CT scans of liver fibrosis patients are more diverse than the CT scans in the public datasets, which were generally without signs of liver fibrosis. Therefore, we fine-tuned the pre-trained V-net by 18 CT scans from liver fibrosis patients. To standardize different external and internal datasets, we normalized the grey values at CT images to [0,1], and resampled the spacing to the same resolution of 3 mm × 3 mm × 2 mm. The best performing fine-tuned V-net was selected at the 47th epoch by a validation set consisting of 5 CT scans from patients of our hospital.

**REFERENCES**

1. Milletari F, Navab N, Ahmadi S (2016) V-Net: fully convolutional neural networks for volumetric medical image segmentation. 2016 Fourth International Conference on 3D Vision (3DV). DOI:10.1109/3DV.2016.79.
